# Distribution and Succession Feature of Antibiotic Resistance Genes Along a Soil Development Chronosequence in Urumqi No.1 Glacier of China

**DOI:** 10.3389/fmicb.2019.01569

**Published:** 2019-07-09

**Authors:** Ju-Pei Shen, Zong-Ming Li, Hang-Wei Hu, Jun Zeng, Li-Mei Zhang, Shuai Du, Ji-Zheng He

**Affiliations:** ^1^State Key Laboratory of Urban and Regional Ecology, Research Center for Eco-Environmental Sciences, Chinese Academy of Sciences, Beijing, China; ^2^College of Resources and Environment, University of Chinese Academy of Sciences, Beijing, China; ^3^College of Animal Science, Yangtze University, Jingzhou, China; ^4^Faculty of Veterinary and Agricultural Sciences, The University of Melbourne, Parkville, VIC, Australia; ^5^School of Geographical Sciences, Fujian Normal University, Fuzhou, China; ^6^Institute of Applied Microbiology, Xinjiang Academy of Agricultural Sciences, Ürümqi, China

**Keywords:** antibiotic resistance gene, glacier retreating, primary succession, horizontal gene transfer, mobile genetic element

## Abstract

Primary succession of plant and microbial communities in the glacier retreating foreland has been extensively studied, but shifts of antibiotic resistance genes (ARGs) with the glacier retreating due to global warming remain elusive. Unraveling the diversity and succession features of ARGs in pristine soil during glacier retreating could contribute to a mechanistic understanding of the evolution and development of soil resistome. In this study, we quantified the abundance and diversity of ARGs along a 50-year soil development chronosequence by using a high-throughput quantitative PCR (HT-qPCR) technique. A total of 24 ARGs and two mobile genetic elements (MGEs) were detected from all the glacier samples, and the numbers of detected ARGs showed a unimodal pattern with an increasing trend at the early stage (0∼8 years) but no significant change at later stages (17∼50 years). The *opr*J and *mex*F genes encoding multidrug resistance were the only two ARGs that were detected across all the succession ages, and the *mex*F gene showed an increasing trend along the succession time. Structural equation models indicated the predominant role of the *intI1* gene encoding the Class 1 integron-integrase in shaping the variation of ARG profiles. These findings suggested the presence of ARGs in pristine soils devoid of anthropogenic impacts, and horizontal gene transfer mediated by MGEs may contribute to the succession patterns of ARGs during the initial soil formation stage along the chronosequence.

## Introduction

The development and dissemination of antibiotic resistance is one of the grand challenges for global public health (WHO, 2014). Majority of previous studies focused on the dissipation and fate of antibiotic resistance genes (ARGs) in habitats influenced by anthropogenic activities, including agricultural soils (Kopmann et al., 2013), heavy metal-contaminated soils (Fahrenfeld et al., 2014; Forsberg et al., 2014), rivers (Ouyang et al., 2015), and animal farms (Zhu et al., 2013). These studies have identified the use of clinically relevant antibiotics in animal husbandry and human medicines as the major factor contributing to the propagation of antibiotic resistance (Levin-Reisman et al., 2017). Growing evidence, however, demonstrates that ARGs are intrinsic in pristine soils with minimal human impacts (Van Goethem et al., 2018), and the natural environment was an important source of ARGs (O’Toole, 2014). Environmental resistome (Wright, 2010) has been associated with ARGs in pathogenic organisms through culture-dependent methods (Poirel et al., 2005) and metagenomic approaches (Forsberg et al., 2012), indicating the potential genetic exchange among soil bacteria and pathogens. The genetic features of antibiotic resistance can be transferred between species through horizontal gene transfer (HGT) mediated by mobile genetic elements (MGEs), which can increase the epidemiological risks of environmental ARGs (Parnanen et al., 2016; Levin-Reisman et al., 2017). Therefore, understanding the prevalence and dissemination of ARGs in natural environments with minimal anthropogenic impacts is essential to unravel the origins and evolution of antibiotic resistance, and may contribute to the prediction of the ARG dispersal risk and the development of mitigation strategies (Martínez et al., 2007; Cytryn, 2013).

Soil is an important reservoir of antibiotics and ARGs (Forsberg et al., 2012). Antibiotics such as beta-lactams, phenicols, tetracyclines, and aminoglycosides are originally derived from soil-resident microbes which have been present in soils for millions of years (O’Toole, 2014). These antibiotics, serving as signaling molecules for communication between microbial cells or as weapons to compete for nutrients and space, would have contributed to the development of antibiotic-resistant bacteria and ARGs in the natural environment (Romero et al., 2011). A number of investigations carried out in remote areas such as permafrost (Mindlin et al., 2008; D’Costa et al., 2011), glacier surface snow, and ice cores (Segawa et al., 2013; Chen et al., 2016), and Antarctic soils and sediments (Tomova et al., 2015), have demonstrated the ubiquity and prevalence of ARGs in these ecosystems. For example, in remote Alaska soils, the *bla*_IMP_ gene encoding resistance to β-lactam was found to be highly abundant (Allen et al., 2009), and the vancomycin resistance element *VanA* was similar to contemporary variants (D’Costa et al., 2011). Despite this progress, however, the succession of ARGs in pristine glacier soils with minimum anthropogenic disturbances, especially under the scenario of increasing retreat of glaciers due to global warming, remains largely unknown.

The forelands of receding glaciers, which have been covered with ice for thousands of years, provide an ideal habitat to study the succession of soil resistome and their associations with soil nutrients and soil microbiome. The glacier retreat is co-occurring with the succession processes of soil nutrients (carbon, nitrogen, phosphorus, etc.) (Zeng et al., 2016), with consequences on the bacterial community, which has been recognized as a major determinant of ARGs (Forsberg et al., 2014). It is widely accepted that microbes are the initial colonizers of retreating glacier foreland area, and microbial activities such as nitrogen fixation, enzymatic activities and respiration, make a major contribution to the nutrients within the foreland (Bradley et al., 2014). In turn, the improvement of nutrition in glacier soils can benefit the growth of microbial communities, which may facilitate the dissipation of ARGs in pristine soils. Therefore, it is imperative to identify the relative contribution of abiotic and biotic factors to the succession patterns of ARGs along the chronosequence of glaciers.

The objective of this study was to assess the successional features of ARGs across a receding glacier foreland in Urumqi Tianshan Mountain No.1 glacier, Xinjiang province, China, based on a space-for-time substitution method. The spatial scale along the retreating glacier foreland spanned a chronosequence of 50 years of soil development. A comprehensive topographic survey of glacier change in this area found the shrinkage of this glacier is mostly related to climate warming (Wang et al., 2016). In this context, we focused here on the succession features of ARGs in the early stages of microbial colonization, during which enzyme activity and nitrogen-cycling microbial communities increased significantly with the succession ages (Zeng et al., 2016). We hypothesized that the changes in microbial community and soil properties along the gradient of succession ages would have consequences on the abundance and diversity of ARGs.

## Materials and Methods

### Sampling Sites

The sampling sites are located in Tianshan Mountain Urumqi No.1 glacier, Xinjiang province, China (43^∘^06′N, 86^∘^49′E). The detailed information of this site has been described previously (Zeng et al., 2016). The annual mean temperature and precipitation are −5.2°C and 645.8 mm, respectively. Observation of Tianshan Mountain No.1 glacier since 1959 has shown rapid glacial retreat due to the increased temperature. In May 2015, we revisited the east branch of Tianshan Mountain No.1 glacier and collected soil samples with 0, 13, 30, 60, 80, 120, 140, and 165 m from the glacier terminus, representing approximate glacial retreating ages of 0 (Surface moraine, SM), 4 (4a), 8 (8a), 17 (17a), 22 (22a), 34 (34a), 40 (40a), and 50 (50a) years (a) (estimated based on a mean glacier retreating rate of 3.5 m year^–1^), respectively (Table 1). At each transect, triplicate soil samples were obtained at an interval of 15–20 m with each mixed with five soil cores at a depth of 0–5 cm. Samples from the SM were set as the beginning of retreating. All samples were shipped directly to the laboratory on ice within 1 day and stored at 4 and −80°C for soil chemical and molecular analysis, respectively.

**TABLE 1:** The sampling sites along the Urumqi No.1 Glacier chronosequence.

**Samples**	**Geographic coordinates**		**Altitude(m)**	**Distance to glacier terminal (m)**
SM	43^∘^07′01″E	86^∘^48′49″N	3785	0
4a	43^∘^07′02″E	86^∘^48′49″N	3789	13
8a	43^∘^07′04″E	86^∘^48′50″N	3789	30
17a	43^∘^07′03″E	86^∘^48′50″N	3889	60
22a	43^∘^07′04″E	86^∘^48′50″N	3803	80
34a	43^∘^07′05″E	86^∘^48′51″N	3809	120
40a	43^∘^07′05″E	86^∘^48′52″N	3823	140
50a	43^∘^07′05″E	86^∘^48′53″N	3821	165

### Soil Chemical Properties

Soil moisture content (H_2_O%) was detected at 105°C, and soil pH was measured with a ratio of 1:2.5 (soil to water) using a pH-meter (Thermo Scientific Inc., Melbourne, Australia). Soil total carbon (TC) and nitrogen (TN) were determined using an elemental analyzer (Vario EL III, Elementar Analysensysteme GmbH, Germany). Soil organic matter (SOM) was measured by the K_2_Cr_2_O_7_ oxidation–reduction colorimetric method. Soil chemical properties are listed in Supplementary Table S1.

### Soil DNA Extraction and High-Throughput Quantitative PCR (HT-qPCR)

Soil total DNA was obtained using the commercial MoBio PowerSoil DNA Isolation Kit (MoBio Laboratories, Carlsbad, CA, United States) following the manufacture’s protocol and was stored at −20°C for the downstream analysis. The DNA quantity was checked using a NanoDrop (ND-1000) Spectrophotometer (Nano Drop Technologies, United States).

The HT-qPCR technique was used to profile a broad variety of ARGs and MGEs on the Bio-Rad CFX384^TM^ Real-Time PCR Detection System (Bio-Rad Laboratories, Hercules, CA, United States) as previously described (Zhu et al., 2017). The array included 296 primers targeting 285 resistance genes for all major classes of antibiotics, and eight transposon-transposase genes, two class 1 integron-integrase genes and the 16S rRNA gene (as the internal control). The targeted ARGs could be categorized into three major resistance mechanisms: cellular protection, antibiotic deactivation, and efflux pumps (Allen et al., 2010; Wright, 2011). A non-template negative control was included in each HT-qPCR run^TM^. Each reaction consisted of 5 μl of SYBR Premix Ex Taq^TM^ (TaKaRa Biotechnology, Dalian, China), 0.5 μl of each primer (10 mM), 1 μl of template DNA, and microbial DNA-free water. The thermal cycling condition includes 10 min at 95°C, subsequently followed by 40 cycles of denaturation at 95°C for 30 s and annealing at 60°C for 30 s. Melting curves were automatically generated by the Bio-Rad CFX Manager software. Three technical replicates were performed for each sample, and negative controls (no DNA template added) were included for each HT-qPCR run. The cycle threshold of 31 was set as the detection limit, and only samples with more than two replicates above the limit of quantification were regarded as positive (Looft et al., 2012). The relative abundance of genes was calculated by normalizing to the bacterial 16S rRNA gene abundance according to a comparative C_T_ method (Schmittgen and Livak, 2008).

The absolute 16S rRNA gene copy numbers were quantified using the primer set Bact1369F/Prok1492R with the Probe^TM^1389F (Suzuki et al., 2000) on the Bio-Rad iQ5 Real-Time PCR Detection System. Each 25 μl reaction system consisted of 12.5 μl Premix Ex Taq^TM^ (Takara Biotechnology, Dalian, China), 10 μM each primer, 5 ng μl^–1^ DNA as template, 1 μl Probe^TM^1389F and nuclease-free PCR-grade water. The plasmid DNA for generating standard curve and amplification profile were used as previously (He et al., 2007). Soil bacterial 16S rRNA gene copy numbers are listed in Supplementary Table S1.

### Bacterial 16S rRNA Gene Sequencing

The glacier soil bacterial community composition across different succession ages was surveyed by prokaryotic 16S rRNA gene Illumina sequencing with the primer pair 515f and 907r (Xu et al., 2012). The 50 μl PCR reaction mixture contained 25 μl of Premix Ex Taq (Takara Biotechnology), 0.4 μl of each primer (10 μM), and 4 μl DNA template (∼10 ng). Amplification conditions were as follows: 94°C for 3 min, followed by six touch-down cycles of denaturation at 94°C (45 s), annealing at 65–58°C (60 s), and expansion at 72°C (70 s), subsequently followed by 22 cycles of 94°C for 45 s, 58°C for 60 s and 72°C for 60 s. The purified PCR products were obtained with a Wizard SV Gel and PCR Clean-Up System (Promega, San Luis Obispo, CA, United States) and then were sent to a local Miseq platform (Illumina, San Diego, CA, United States) at Novegene, Beijing, China.

The obtained raw sequences were split by samples, quality filtered and de-noised using Quantitative Insights Into Microbial Ecology (QIIME) according to the operation procedure (Caporaso et al., 2010) with less than eight homopolymers. The sequencing depth was at least 50,000 reads for each sample. The resultant high-quality reads with more than 300 bp length were classified with the operational taxonomic units (OTUs) at a 97% similarity using UPARSE clustering (Edgar, 2013). The taxonomic assignment of representative sequences was achieved using the Ribosomal Database Project (RDP) Classifier (Wang et al., 2007) with a minimum confidence of 80%. To correct the difference in the sequencing efforts, sequences were rarefied to 20,000 reads among all the samples before the downstream analysis. Bacterial sequences obtained in this study are accessible through the EMBL accession number PRJEB20522.

### Statistical Analysis and Data Visualization

One-way analysis of variance (ANOVA) followed by the Student-Newman–Keuls test was carried out to compare the difference in diversity and the relative abundances of ARGs across different succession ages in SPSS 22.0. *P* < 0.05 was considered to be statistically significant. Spearman correlation test was performed to assess the correlations between the relative abundance of ARGs and MGEs across the succession ages. The heat map of individual ARGs was generated from the relative abundance of ARGs using Origin Pro 2016. Non-metric multidimensional scaling (NMDS) ordinations based on Bray–Curtis distances were carried out to check the shift of ARG profiles along the chronosequence using the “vegan” package with 999 permutations in R.

Co-occurrence networks were constructed by obtaining Spearman correlation coefficients (ρ) from the bacterial relative abundance data at the order level against all ARGs detected along the succession ages (Hu et al., 2016). ARGs occurred in less than six samples and were excluded in the network analysis. Correlations with rho coefficients greater than ρ = 0.6, or below ρ = −0.6, and with significant adjusted *P* values (*P* < 0.05) were selected in the analysis. Statistical significance was demonstrated through a permutation testing procedure. Cytoscape v.3.6.1 was chosen for network visualization.

The structural equation model (SEM) was established to assess the direct and indirect effects of bacterial abundance and community composition (the relative abundance of OTU), *intI1* (MGEs), and soil properties (including pH, TN, TC, SOM, and C/N ratio) on the ARGs’ diversity (number of detected ARGs). Bacterial community compositions or soil properties were firstly processed with principal component analysis using SPSS 20 (SPSS Inc., Chicago, United States) and the first coordinate was extracted for further correlation analysis. The pairwise correlations were further determined among these variables using SPSS 20, and the standardized data using *Z*-scores were imported into AMOS 21 (SPSS Inc., Chicago, United States) for SEM construction using the maximum-likelihood estimation (Hu et al., 2016). The models meet multiple goodness-of-fit criteria: non-significant χ2 test (*P* > 0.05), a root mean square error of approximation (RMSEA) less than 0.08, a goodness-of-fit index (GFI) higher than 0.90, and an Akaike information criterion (AIC) from the default model lower than that from the saturated model and the independence model. The standardized total effects of each factor on ARGs diversity were calculated by summing all direct and indirect pathways between the factor and ARGs.

## Results

### Diversity of ARGs and MGEs Along the Glacier Chronosequence

**FIGURE 1:**
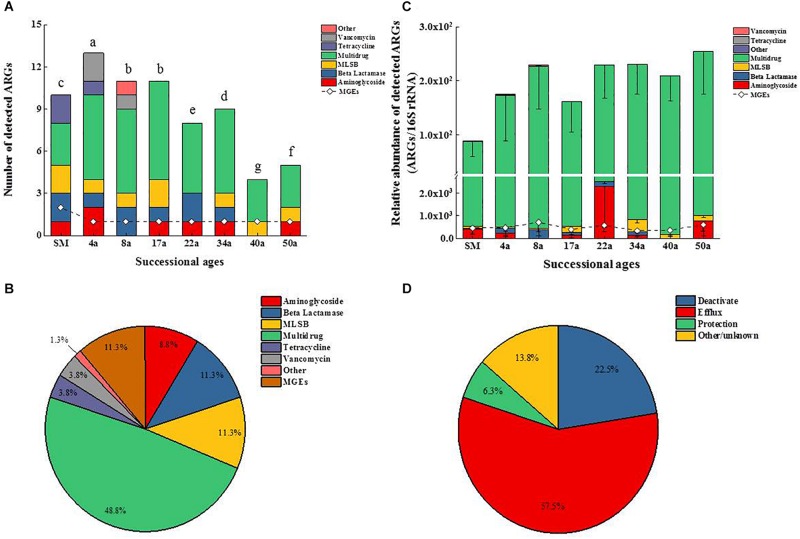
Changes of ARGs and MGEs in the detected number **(A)** and the relative abundance **(B)**. Classification of ARGs based on the antibiotic to which they confer resistance **(C)** and resistance mechanisms **(D)** along the glacier chronosequence. MLSB, Macrolide–Lincosamide–Streptogramin B resistance; MGEs, mobile genetic elements. Successional ages representing approximate glacial retreating ages of 0 (SM), 4 (4a), 8 (8a), 17 (17a), 22 (22a), 34 (34a), 40 (40a), and 50 (50a) years. The difference in the number of detected ARGs across succession ages in **(A)** was assessed by a one-way analysis of variance (ANOVA) followed by Student–Newman–Keuls test. Different lowercase letters on the bar indicate significant difference (*P* < 0.05). No letters labeled on the bar in **(C)** means no significant difference in the relative abundance of ARGs across succession ages.

The HT-qPCR array detected a total of 26 genes (including 24 ARGs, one transposase genes, and one class 1 integron-integrase gene) from the soil samples. The numbers of detected ARGs ranged from 5 to 14 across the chronosequence and tended to slightly decline with increasing succession ages (Figure 1A). Among all the soil samples, the highest and lowest numbers of detected ARGs were recorded in the ages of 4 and 40 a soil samples, respectively.

**FIGURE 2:**
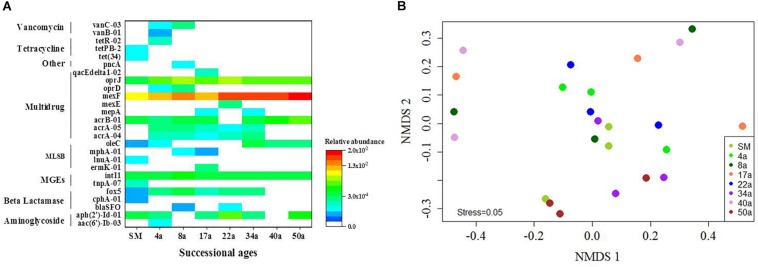
The distribution profiles of ARGs across the succession ages. **(A)** A heat map of the ARGs along the successional ages. Values plotted are the ΔCt with 16S rRNA of each sample. Red and blue colors indicate high and low relative abundances, respectively, while white indicates not detected. **(B)** The NMDS analysis based on the relative abundance of ARGs using Bray–Curtis distances. Circle symbols with different colors represent approximate glacial retreating ages of 0 (SM), 4 (4a), 8 (8a), 17 (17a), 22 (22a), 34 (34a), 40 (40a), and 50 (50a) years. MLSB, Macrolide–Lincosamide–Streptogramin B resistance; MGEs, mobile genetic elements.

The multidrug resistance genes were the most frequently detected (48.8%), followed by the genes encoding resistance to β-lactam (11.3%), macrolide–lincosamide–streptogramin B (MLSB, 11.3%), aminoglycoside (8.8%), tetracycline (3.8%), and vancomycin (3.8%) (Figure 1C). Genes conferring resistance to sulfonamides and quinolones were not detected. The ARGs detected in soil samples potentially conferred resistance to most major classes of antibiotics and encompassed three major resistance mechanisms: efflux pumps (57.5%), antibiotic deactivation (22.5%) and cellular protection (6.3%) (Figure 1D). For the MGEs, the class 1 integron-integrase *intI1* gene as a marker gene for HGT potential was detected across all the succession ages (Figure 2A). The IS*Ecp1B* family transposase gene (*tnpA-07*) was only detected in SM samples (Figure 2A).

### The Relative Abundance of ARGs and MGEs Along the Glacier Chronosequence

**FIGURE 3:**
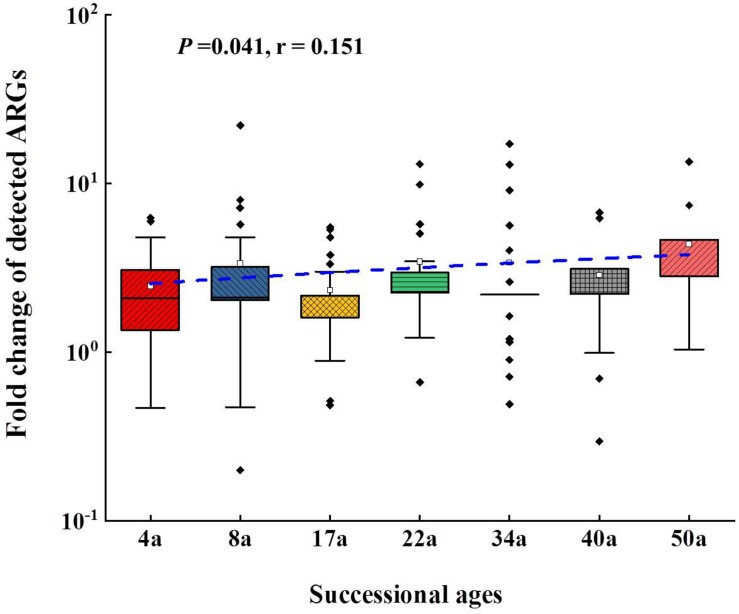
The fold changes of the detected ARGs compared to surface moraine (SM). Line regressions between fold changed of detected ARGs and succession ages are shown as a blue line. In the box plots, the symbols indicate the following: box, 25th to 75th percentile; horizontal line, median; whiskers, 10th and 90th percentile; and square, maximum value. The sample from SM was set as reference. The *t* test showed that there was significant enrichment of ARGs following the succession.

The relative abundance of ARGs calculated by normalizing against the bacterial 16S rRNA gene showed different patterns compared with that of the numbers of detected ARGs across the succession ages. It showed an increasing trend in the early ages (0∼8 years) but maintained largely unchanged in the later ages (Figure 1B). Multidrug resistance genes were the most abundant ARG types, followed by aminoglycoside, β-lactam and MLSB. Among all the 26 detected ARGs, only *opr*J and *mex*F were found in all the soil samples with consistently higher relative abundance, ranging from 6.07 × 10^–3^ to 3.25 × 10^–4^ and 1.97 × 10^–2^ to 7.70 × 10^–3^, respectively (Figure 2A). The relative abundance of the *mex*F gene showed an increasing trend along the succession ages. The NMDS analysis revealed that there is no clear pattern of ARG profiles from glacier foreland soils across succession ages (Figure 2B). The *intI1* gene encoding class 1 integron-integrase accounted for 80–100% of the relative abundance of total MGEs. The fold changes of ARGs and the relative abundance of MGEs in aged soil samples compared to SM showed a significantly increasing trend along the succession ages (*P* < 0.05, Figure 3). Bacterial 16S rRNA gene copy numbers showed a decreasing trend along the succession age (*P* < 0.05), which had a significant correlation with TC (*r* = 0.717, *P* < 0.001).

### Correlation Analysis

**FIGURE 4:**
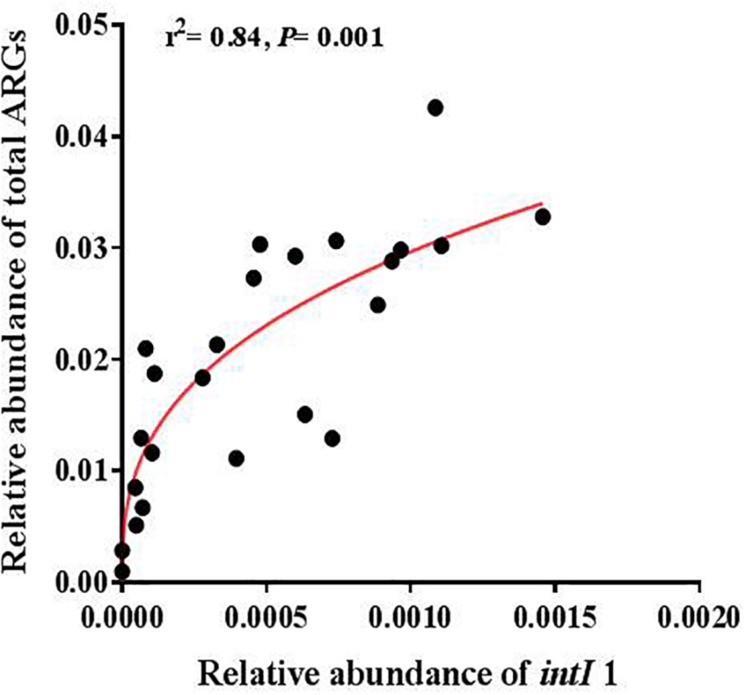
The relative abundance of the *intI1* gene was positively correlated with the relative abundance of total ARGs.

A significantly positive correlation was found between the abundance of total ARGs and total MGEs (*r* = 0.644, *P* < 0.001). The changes of MGEs in the glacier soil samples were also positively correlated with some specific classes of ARGs, including β-lactamase (*P* < 0.01) and multidrug (*P* < 0.01). The total abundance of ARGs and seven specific ARGs had a significantly positive correlation with the *intI1* gene (Figure 4 and Table 2).

**TABLE 2:** The correlations between the relative abundance of *intI1* and seven ARGs.

**ARGs**	**Class**	**Spearman’s correlation coefficient**
*acr*A-04	Multidrug	0.52^∗∗^
*acr*A-05	Multidrug	0.50^*^
*acr*B-01	Multidrug	0.49^*^
*aph*(2′)-Id-01	Aminoglycoside	0.57^∗∗^
*fox*5	Beta Lactamase	0.49^*^
*mex*F	Multidrug	0.80^∗∗^
*opr*J	Multidrug	0.85^∗∗^

^*^P < 0.05, ^∗∗^*P* < 0.01.

**FIGURE 5:**
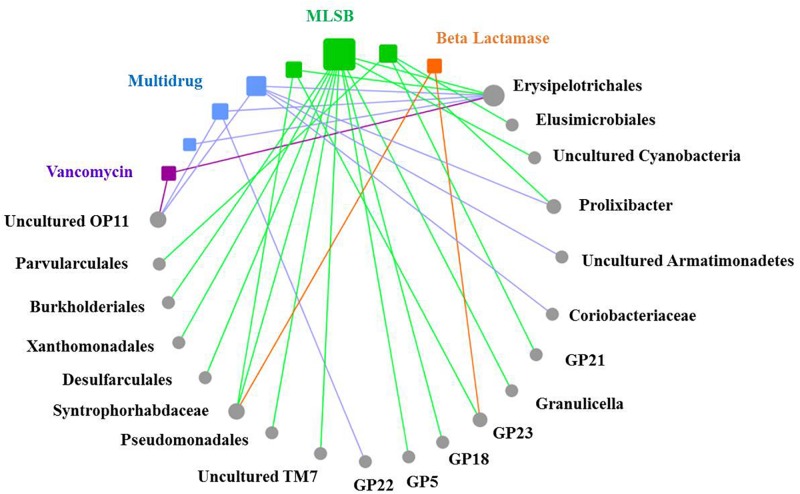
The co-occurrence patterns between ARG types and bacterial taxa at the order level along the succession ages. The nodes with different colors mean different ARG types (squares) and bacterial taxa (circles), and the edges correspond to strong (correlation coefficients |ρ| > 0.6) and significant (*P* < 0.05) correlations between nodes. Statistical significance is preformed through a permutation testing procedure. Bacterial taxonomical information at the order level is labeled for each node. The size of each node is proportional to the number of significant correlations between nodes.

**FIGURE 6:**
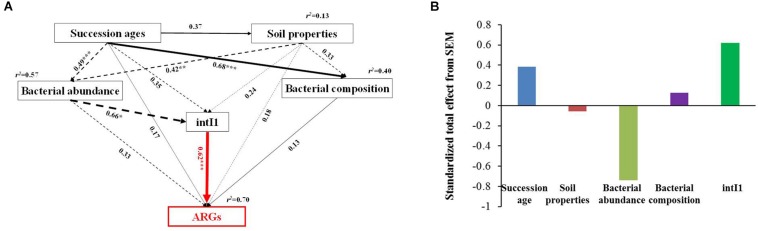
Structural equation models showing the direct and indirect effects of succession ages, soil properties, MGEs, bacterial abundance, and community composition on the ARG diversity in the glacier samples **(A)**. Standardized total effects (direct plus indirect effects) derived from the structural equation models **(B)**. Continuous and dashed arrows show positive and negative relationships, respectively. Numbers adjacent to arrows are path coefficients, and width of the arrows is proportional to the strength of path coefficients. The response variable (ARG diversity) is highlighted in red. The hypothetical models fit our data well, as demonstrated by χ2 = 1.57, *P* = 0.46, GFI = 0.98, AIC = 38.26, and RMSEA = 0.00. *R*^2^ values indicate the proportion of variance explained for each variable. Significance notes are indicated via the following: ^*^P < 0.05, ^∗∗^*P* < 0.01, and ^∗∗∗^*P* < 0.001.

Based on the network analysis, eight ARGs were found to have significant correlations with their possible hosts (Figure 5). In this network, multidrug and MLSB resistance genes showed more intensive connections with the bacterial communities. SEMs were constructed to identify the direct and indirect effects of succession ages, soil properties, bacterial communities, and MGE (*intI1*) on the ARG distribution along the chronosequence (Figure 6A). Our SEMs explained 70% of the variance in the changes of ARGs. The *intI1* gene (belonging to MGEs) was found to have significantly positive influences on the ARG distribution (λ = 0.62, *P* < 0.001). Succession ages had significantly negative effects on the bacterial abundance (λ = 0.49, *P* < 0.001) and positive effects on the bacterial composition (λ = 0.68, *P* < 0.001). The bacterial abundance had significantly negative effects on MGEs (λ = 0.66, *P* < 0.05), but no significant influences on ARGs. Overall, ARG patterns were directly influenced by the *intI1* gene, followed by succession ages, while the bacterial abundance showed minimal contributions (Figure 6B).

## Discussion

Glacier forelands, as a newly exposed terrestrial ecosystem, are considered as an ideal habitat to study the origin and evolution of ARGs (Van Goethem et al., 2018). In this study, in total 24 ARGs were detected in glacier forelands, which is significantly lower than that detected in anthropogenically disturbed environments, such as swine farms (Zhu et al., 2013) and maize field (Hu et al., 2016) using the same HT-qPCR technique. ARGs are detected from all the succession ages, indicating that antibiotic resistance is rife in remote natural environments (O’Toole, 2014). Previous studies have demonstrated that ARGs are widely distributed in the environments with less human impacts, including Arctic permafrost (Perron et al., 2015), pristine Antarctic soils (Van Goethem et al., 2018), and Alaskan soils (Allen et al., 2009). A multitude of studies have shown that most native soil bacteria harbored high levels of ARGs and have been present for millions of years in the pre-antibiotic era (Aminov and Mackie, 2007). ARGs could also be transferred to remote areas by airborne bacteria or migrant birds as demonstrated by a global investigation on snow samples (Segawa et al., 2013). Furthermore, antibiotics can be produced by soil indigenous fungi and bacteria, which will exert selective pressure on soil resident microbes (Allen et al., 2010). All these studies suggested the high presence of ARGs in the habitats with less anthropogenic impact, providing a potential reservoir of ARGs that may be helpful in tracking the evolution of resistant pathogens (Van Goethem et al., 2018).

The diversity and the relative abundance of ARGs showed an increasing trend along the chronosequence in early stages (0∼8 year), while they decreased or remained unchanged in later stages (17∼50 years). The unimodal pattern of ARGs followed a similar trend as observed for plants (Jones and Henry, 2003) and microbes (Tian et al., 2017). The possible reason responsible for the relative abundance distributions may be due to the competition of invaders for the available resources, contributing to the deviations from linearity (Rivett et al., 2016). The composition and diversity of ARGs in later stages are mainly driven by MGEs through the HGT pathway, as the SEM analysis showed that MGEs (the *intI1* gene in this study) are the major contributors to the variation of ARG diversity. This was further confirmed by the co-occurrence network analysis between bacterial taxa and ARGs. For instance, the most dominant ARGs, i.e., multidrug and MLSB resistance genes, had more connections with microbial taxa, indicating some ARGs might be carried by multiple bacterial phyla. The highly shared ARGs between different hosts are mainly attributed to HGT mediated by MGEs, favoring the distribution of ARGs in the environment (Hu et al., 2016). Furthermore, significant correlations were found between the relative abundance of total ARGs and the *intI1* gene, which is consistent with previous findings that the propagation of ARGs is more closely linked with MGEs (Wang et al., 2014; Klumper et al., 2015). The SEM analysis showed a minor impact of the bacterial community on ARG patterns in contrast with the previous reports of the bacterial phylogenetic structures as a major determinant of ARGs (Forsberg et al., 2014). Although microbes are widely considered as the initial colonizers of recently exposed glacier retreating soils, the bacterial abundance showed a decreasing trend along the succession ages, which was largely driven by the changes in soil nutrient concentrations (e.g., carbon). The apparent decrease in carbon and nitrogen along the chronosequence sequences, especially in progressed successional ages, may provide evidence for the changes (Jones and Henry, 2003). The findings suggested that in the earliest stages of glacier succession, MGEs might contribute to the acquisition of resistance in extreme environments (Hu et al., 2017).

As a marker gene of MGEs, the *intI1* gene is an indispensable part of integrons, which are natural capture systems. More than 15% of bacterial species were detected to carry integron elements by the genome-sequencing technology (Yan et al., 2007; Cambray et al., 2010). In our study, the *intI1* gene was detected with high relative abundance along the chronosequences, indicating its potential role in the persistence and evolution of ARGs in undisturbed soils (Partridge et al., 2009). Integrons are widely distributed not only in temperate environments such as forest soils and riverine sediments, but also in extreme habitats, including Antarctic soils and hot springs (Stokes et al., 2001; Michael et al., 2004; Gillings et al., 2009; Elsaied et al., 2010). With the aid of MGEs, HGT is a main pathway for relocating the same ARG in different microbial hosts, facilitating the propagation of ARGs (Gillings et al., 2009). MGEs such as integrons, transposons and plasmids have been well recognized for their contribution to the acquisition and dissemination of ARGs via HGT (Strumper et al., 2005).

Although a general decreasing trend was recorded along the chronosequence, we found that individual genes including ARGs and MGEs showed different patterns, with some of them remaining unchanged while others showed a substantial increase in abundance during the glacier retreating process. A noticeable example is the *mex*F gene, which was significantly correlated with succession ages (*r* = 0.390, *P* < 0.05). Additionally, the *opr*J and *mex*F genes encoding multidrug resistance were the only two ARGs that were detected across all the succession ages, and significantly correlated with the *intI1* gene, suggesting these two genes are of ancient inheritance and may have transferred between cells or species through the pathway of HGT. A similar higher abundance of the *mex*F gene was also recorded in other environmental habitats such as landfills (Liu et al., 2018), agricultural ecosystems (Xiao et al., 2016), and aquatic systems (Gao et al., 2018). Elevated abundance of the *mex*F gene was closely related with the succession feature of *Pseudomonas* carrying multidrug resistance genes, as confirmed by the significant correlation between the relative abundance of the *mex*F gene and *Pseudomonas* (*r* = 0.431, *P* < 0.05). This is consistent with other studies that the prevalence of *Pseudomonas* is largely responsible for variation of the *mex*F gene (Jia et al., 2015; Liu et al., 2018). ARGs conferring multidrug resistance were found as the dominant types in this study, and the efflux pump is the main resistance mechanism. Efflux pump genes are widely present in all microorganisms (Allen et al., 2010), especially those in extreme environments (Chen et al., 2013), such as glacier soils under nutrient depleted conditions in this study. Furthermore, efflux pumps have broad specificity (Piddock, 2006), suggesting that these efflux pump genes are ancient intrinsic genotypes that predate the human use of antibiotics.

The β-lactamases conferring resistance to β-lactam antibiotics were reported to be abundant in human impacted soils (Hu et al., 2016) and undisturbed soils (Allen et al., 2009). However, β-lactamases were detected with low abundance in the glacier samples in this study. Our results are consistent with the recent findings from the Mackay Glacier region soil samples using the shotgun metagenomics method (Van Goethem et al., 2018), in which a very low abundance of genes encoding β-lactam resistance was found. A global investigation of snow samples also demonstrated the absence of the β-lactam gene in Greenlandic and Alaskan glacier samples (Segawa et al., 2013).

## Conclusion

This is the first study showing the dynamics of ARGs along an early primary glacier chronosequence. It was confirmed that ARGs have been present in the natural environment long before the use of antibiotics. Changes of ARGs demonstrated a unimodal pattern with increasing trend at early stages (0∼8 years) with no significant change at later stages (17∼50 years). These results also suggested that edaphic properties and succession ages greatly affected the bacterial community abundance, and MGEs made a substantial contribution to the patterns of ARGs along the chronosequence. This succession feature of ARGs along the glacier retreating process provides insight into the evolution process of native ARGs and the potential contribution of soil resistome to clinically related antibiotic resistance.

## Data Availability

The datasets generated for this study can be accessed from EMBL, PRJEB20522.

## Author Contributions

J-PS and J-ZH conceived the study. Z-ML, JZ, and J-PS performed the experiments. J-PS, Z-ML, and SD analyzed the data. J-PS, Z-ML, H-WH, L-MZ, and J-ZH wrote the manuscript.

## Conflict of Interest Statement

The authors declare that the research was conducted in the absence of any commercial or financial relationships that could be construed as a potential conflict of interest.
