# Supplementary material for: Distribution and Succession Feature of Antibiotic Resistance Genes Along a Soil Development Chronosequence in Urumqi No.1 Glacier of China
Source: Front Microbiol. 2019 Jul 9;10:1569. doi: 10.3389/fmicb.2019.01569 (PMC6629927; doi:10.3389/fmicb.2019.01569)
Supplement: Supplementary file 1 [file Table_1.DOCX]

**Table S1. The Urumqi No.1 glacier soil chemical properties and bacterial abundance**

| Samples | Moisture  (%) | pH | TN  (g·kg^-1^) | TC  (g·kg^-1^) | C/N | OM  (g·kg^-1^) | Bacterial  16S rRNA |
| --- | --- | --- | --- | --- | --- | --- | --- |
| SM | 10.09 | 7.06 | 0.49 | 6.75 | 13.78 | 1.92 | 6.89x10^9^ |
| 4a | 7.88 | 7.64 | 0.68 | 5.79 | 8.51 | 1.20 | 9.03 x10^9^ |
| 8a | 8.06 | 7.70 | 0.57 | 5.38 | 9.44 | 1.19 | 7.67 x10^9^ |
| 17a | 7.94 | 7.56 | 0.40 | 5.26 | 13.15 | 1.08 | 4.23 x10^9^ |
| 22a | 7.11 | 7.49 | 0.39 | 5.33 | 13.67 | 1.16 | 3.99 x10^9^ |
| 34a | 5.79 | 7.64 | 0.44 | 5.32 | 12.09 | 0.87 | 4.21 x10^9^ |
| 40a | 5.77 | 7.69 | 0.45 | 5.65 | 12.56 | 1.01 | 3.41 x10^9^ |
| 50a | 6.01 | 7.50 | 0.38 | 5.37 | 14.13 | 1.27 | 1.72 x10^9^ |

The data shown in the table are mean values of the three replicates

Abbreviations: TC, Total carbon; TN, Total nitrogen; C/N, the ratio of total carbon to total nitrogen; OM, organic matter

Unit for bacterial 16S rRNA gene: copy numbers per g dry soil
